# The extent of energy drink marketing on Canadian social media

**DOI:** 10.1186/s12889-023-15437-w

**Published:** 2023-04-25

**Authors:** Chanelle Ayoub, Meghan Pritchard, Mariangela Bagnato, Lauren Remedios, Monique Potvin Kent

**Affiliations:** School of Epidemiology and Public Health, Faculty of Medicine, University of Ottawa, 600 Peter Morand Cres., Room 301J, Ottawa, ON K1G 5Z3 Canada

**Keywords:** Caffeinated energy drinks, Adolescent, Children, Beverage marketing, Social media, Viral marketing, Celebrity endorsement, Health appeals, Cross-promotions

## Abstract

**Background:**

Caffeinated energy drink (CED) consumption among children and adolescents is a growing global public health concern due to its potential to produce adverse effects. CED marketing viewed by children and adolescents contributes to this problem as it increases consumption and favourable attitudes towards these high-caffeine and high-sugar products. This study aimed to describe the social media marketing of CEDs by estimating the frequency of user-generated and company-generated CED marketing and analyzing the marketing techniques used by Canadian CED brands on social media.

**Methods:**

CED products and brands were identified using the list of CEDs that received a Temporary Marketing Authorization from Health Canada in June 2021. The data on the frequency, reach and engagement of CED-related posts created by users and Canadian CED brands on Facebook, Instagram, Twitter, Reddit, Tumblr, and YouTube were licensed from Brandwatch for 2020–2021. A content analysis was conducted to assess the marketing techniques used in Canadian CED company-generated posts using a coding manual.

**Results:**

A total of 72 Canadian CED products were identified. Overall, there were 222,119 user-level mentions of CED products in total and the mentions reached an estimated total of 351,707,901 users across platforms. The most popular product accounted for 64.8% of the total user-level mentions. Canadian social media company-owned accounts were found for 27 CED brands. Two CED brands posted the most frequently on Twitter and accounted for the greatest reach, together making up 73.9% of the total company-level posts and reaching 62.5% of the total users in 2020. On Instagram/Facebook, the most popular brand accounted for 23.5% of the company-level posts and 81.3% of the reach between July and September 2021. The most popular marketing techniques used by Canadian CED brands were the use of viral marketing strategies (82.3% of Twitter posts and 92.5% of Instagram/Facebook posts) and the presence of teen themes (73.2% of Twitter posts and 39.4% of Instagram/Facebook posts).

**Conclusion:**

CED companies are extensively promoting their products across social media platforms using viral marketing strategies and themes that may appeal to adolescents. These findings may inform CED regulatory decision-making. Continued monitoring is warranted.

## Introduction

Caffeinated energy drinks (CEDs) are popular among Canadian youth due to their perceived ability to improve mental or physical performance and are often consumed within social and sport settings [1, 2]. CEDs are the fastest growing products in the beverage industry and are becoming increasingly prevalent among youth in recent years [3, 4]. A Canadian study found that 74% of youth between the ages of 12 and 24 years old have consumed an energy drink [5]. Among Ontario high school students, over 18% reported consuming CEDs weekly [6]. CED consumption in this population is a growing global public health concern due to their potential to produce adverse effects and its association with risk-taking behaviours in adolescents [7–10]. Recent studies have reported adverse events associated with CED consumption in individuals between 11 and 19 years old, with insomnia, stress, and depressive mood being most frequent [11]. Serious adverse cardiovascular and neuropsychiatric outcomes in this population have also been reported, including mood disorders, seizures, gastrointestinal issues and even death [11–13]. In a Canadian study, 55.4% of youth reported experiencing an adverse event related to their CED consumption in the past [14].

Caffeine is the most common ingredient in CEDs and is primarily associated to CED-related adverse events due to caffeine toxicity in high doses [13]. CEDs also contain several other ingredients that may produce cardiovascular effects and interact synergistically with caffeine, such as taurine, herbal extracts, B vitamins, and minerals, which may further increase the risk of toxicity [13, 15, 16]. Additionally, the sugar content in CEDs is comparable to traditional sugar-sweetened beverages and soft drinks, which presents additional health implications, particularly for childhood obesity [3, 10].

### CED marketing in Canada

CED marketing targeting children and adolescents is concerning, as it may increase the positive attitudes and preferences, as well as consumption of these harmful products [17–20]. CED marketing regulations vary between counties, and ranges from widespread restrictions to less stringent policies. In United States (US), the Food and Drug Administration does not impose any restrictions on ingredients, labelling, or marketing activities of CED products, as CEDs are regulated as dietary supplements [3, 21]. Within the European Union (EU), CED labels must include a warning statement for high caffeine content and for not being recommended to children, followed by the exact amount of caffeine present [21]. Although most EU countries have made voluntary commitments to restrict CED marketing to children, certain countries, such as Denmark, prohibit the sales of CEDs altogether [21, 22]. In Canada, under the Food and Drugs Act (FDA), companies that manufacture CEDs must receive a Temporary Marketing Authorization to market their product under specific conditions and require presenting a caution statement on the packaging warning against child consumption [15, 23]. In addition, CED marketing to children under 12 years old is prohibited [23]. The Canadian Beverage Association (CBA) has also pledged to restrict the marketing of CEDs to children under 12 years old under the Energy Drinks Marketing Code [24]. However, the regulations by government and industry have limited effectiveness, as children under 12 years old continue to be exposed to CED advertisements in their environment, on television and online [25–27].

### Digital marketing

In Canada, 55% of children (10–13 years old) and 77% of adolescents (14–15 years old) owned a smartphone in 2018 [28]. The most popular function of a smartphone among adolescents is to use social media, such as YouTube, Snapchat, Twitter, Instagram and Facebook [29]. In 2017, 33% and 25% of Ontario adolescents respectively spent 1–2 h and 3–4 h a day on social media, with 20% spending over five hours a day [30]. Unsurprisingly, the food and beverage industry is increasingly shifting to digital media as the main platform for advertising to children and adolescents via websites and social media [31]. However, no research has examined the frequency and content of CED marketing on social media in the Canadian context.

The objectives of this study are to estimate the frequency of user-generated CED marketing and engagement with CED brands, to estimate the frequency of company-generated CED marketing on Canadian social media and to analyze the marketing techniques used by CED brands on social media.

## Methods

### Identifying CED products and brands

The CED products and brands of interest were identified using the list of CEDs that received a valid Temporary Marketing Authorization letter from Health Canada in June 2021 [32]. Both regular and diet versions of CEDs were included, however energy shots (which are regulated as Natural Health Products in Canada) and different flavours of the same product were excluded. A final list of 72 unique products was created for analysis.

### Data collection

The data on the frequency and reach of CED advertisements on Canadian social media as well as the engagement with Canadian CED brands were licensed from Brandwatch for 2020–2021. Brandwatch is a leading digital consumer intelligence platform and a social media monitoring tool that can be used to track insights and interactions with brands and audiences [33]. Search queries were created to assess the frequency and reach of posts on Twitter, Instagram, Facebook, Reddit, YouTube, and Tumblr. Two different methodologies were used. One methodology was used to estimate the user-level engagement and mentions of CED products (user-generated analysis). In this study, engagement is specifically defined as the total number of impressions on Twitter posts containing a mention of a CED product (i.e., retweets, searches, and views on a post) and total followers of all Twitter users who have posted about a relevant product. At the user-level, Brandwatch refers to all mentions of CED products found in social media posts based on the search queries as “mentions”. The second methodology was used to estimate the frequency and engagement with the posts generated by a company’s social media account (company-generated analysis).

### User-generated analysis

Queries were created to track posts by all users published from January 1, 2020 to December 31, 2020 on Twitter, Reddit, Tumblr, and YouTube. Instagram and Facebook were not included in the user-generated analysis, as they required a separate methodology due to separate licensing agreements between Brandwatch and these platforms. User-generated marketing is defined as the content created by all social media users and the conversations surrounding a given product. All mentions collected by Brandwatch (i.e., the total number of posts in the user-level analysis), including any posts by company accounts and social media influencers, were included in this analysis, as their posts equally reflect social media conversations regarding CED products.

The queries consisted of keywords relevant to CED brands and their products, as well as operator words (e.g. AND, OR) to detect relevant posts. The product name was the first keyword included in the query, as written in Health Canada’s list of CED products. If the term “energy drink” was not included in the product name according to the Health Canada list, it was added. In addition, other common or shortened names, spelling variations, colloquial terms, relevant hashtags, and the company name (if different from the product name) were added to the queries. Geographical location tags (geotags) that exclude other countries were also added to the queries, as only data from Canada were included in the analysis.

After searching the queries, the outcome measures for each social media platforms (excluding Facebook and Instagram) were extracted as follows: frequency of mentions (i.e. the sum of all social media posts containing CED product name); total reach (i.e. the estimated number of people who have seen the CED mentioned); and gender of the user mentioning the CED (if information was publicly available). On Twitter only, the following two outcomes were also examined: total impressions (i.e., the number of retweets, searches, and views on a post) and total followers (i.e., the sum of all followers of all Twitter users who have posted about a relevant product).

### Company-generated analysis

To assess the company-level marketing of CED products and brands on Canadian social media, a list of Canadian CED social media accounts for Twitter, Instagram and Facebook was created in June 2021. Social media accounts were searched via the CED company websites and only Canadian accounts were included in the analysis. Other social media platforms (Tumblr and Reddit) were not included in the analysis as companies generally do not have accounts on these platforms. YouTube was not included due to the limitations in Brandwatch’s access to company-level data on this streaming platform.

The account names (handles) were inputted into Brandwatch queries to collect company-generated data. For Twitter, data were collected from January 1, 2020, to December 31, 2020. Brandwatch does not have access to historical data on Facebook and Instagram accounts, as such it was only possible to collect data for July, August, and September 2021 on these platforms.

For Twitter, the following company-level outcome measures were obtained from the Brandwatch queries: frequency of mentions, total reach, total impressions, and total followers. For Instagram and Facebook, the data were combined for both these platforms and frequency of mentions and total reach were examined.

### Content analysis

A content analysis of marketing techniques was conducted on relevant company-generated posts published in 2020 (Twitter only) and from July 1, 2021, to September 30, 2021 (Facebook and Instagram only). Posts that only contained French content were excluded, as only English content was assessed and coded. For companies that exclusively sell CED products, all company-published posts were coded. For companies that do not exclusively sell CED products, only posts that marketed their CED products were coded. If there were no CED-related posts on the company account, the brand was excluded from the company-level analysis.

The marketing techniques found in posts published by Canadian CED company accounts on Twitter, Facebook, and Instagram were coded by a single reviewer (CA) who received training to use the coding manual (by LR and MB). Any uncertainty when coding the content was resolved through discussion with LR and MB. The content of the posts were coded using a coding manual adapted from previous research that assessed marketing techniques that may appeal to children and adolescents, as well as general social media marketing techniques [34, 35].

Using the coding manual, audio and visual techniques, such as animation, special effects and music or jingles were identified. The child-related techniques that were examined included: the presence of a child product (e.g., candy); presence of children; child language (e.g., “hey kids!”); child themes (e.g., fantasy, magic, virtual worlds); licensed characters; spokes characters (i.e. characters owned by brands); child incentives (e.g., free gifts, toys, books); and parent-child situations. The teen-related techniques were examined included: the presence of teens; teen language (e.g., “hey dude” or other slang); and teen themes (e.g., sports or extreme sports, risk-taking, popular music/culture, video games). Other techniques that may appeal to a variety of age groups were also assessed including: celebrity endorsements; cross-promotions that feature movies, events, video games, or other references, purchase incentives (i.e., contests/giveaways); interactive elements, which include call-to-action online (i.e., directing users to brand website), viral marketing strategies (i.e., hashtags, tagging, and encouraging users to comment, like and share), games or polls, and sharing user-generated content; and ad appeals (e.g., convenience, economical, health appeal).

Frequency tables were created for all outcomes based on timelines and social media platforms.

## Results

### User-generated analysis

The total number of mentions and total reach for each product on Twitter, Reddit, Tumblr, and YouTube for 2020 are shown in Table 1. Overall, CED products were mentioned a total of 222,119 times in user-generated posts (i.e., mentions) and these mentions reached a cumulative estimated total of 351,707,901 users. The most popular product was mentioned 143,834 times, making up 64.8% of the total mentions. In general, more men than women posted about CED products (mentions) and were more likely to see a post about CED products (estimated reach). Among the posts that contained gender information, men accounted for 72.5% (56,682) of the mentions and 72.4% (59,139,856) of the estimated reach (data not shown).

**Table 1:** Frequency of CED user-generated mentions on all platforms^a^ and total reach of posts in 2020

Products	Total Mentions n (%)	Total Reach n (%)
**Product 1**	143,834 (64.8%)	220,051,010 (62.6%)
**Product 2**	22,156 (10.0%)	68,024,150 (19.3%)
**Product 3**	21,513 (9.7%)	16,077,402 (4.6%)
**Product 4**	11,731 (5.3%)	11,869,418 (3.4%)
**Product 5**	5,916 (2.7%)	9,847,132 (2.8%)
**Product 6**	4,348 (2.0%)	6,566,236 (1.9%)
**Product 7**	2,814 (1.3%)	5,565,852 (1.6%)
**Product 8**	1,996 (0.9%)	2,275,567 (0.6%)
**Product 9**	1,624 (0.7%)	1,390,039 (0.4%)
**Product 10**	1,044 (0.5%)	3,353,772 (1.0%)
**Product 11**	1,022 (0.5%)	722,388 (0.2%)
**Product 12**	676 (0.3%)	465,246 (0.1%)
**Product 13**	594 (0.3%)	1,430,362 (0.4%)
**Product 14**	392 (0.2%)	401,479 (0.1%)
**Product 15**	383 (0.2%)	495,459 (0.1%)
**Product 16**	218 (0.1%)	135,510 (0.0%)
**Product 17**	218 (0.1%)	325,479 (0.1%)
**Product 18**	204 (0.1%)	840,959 (0.2%)
**Product 19**	203 (0.1%)	107,083 (0.0%)
**Product 20**	179 (0.1%)	1,190,639 (0.3%)
**Product 21**	154 (0.1%)	32,761 (0.0%)
**Product 22**	132 (0.1%)	94,283 (0.0%)
**Product 23**	129 (0.1%)	63,489 (0.0%)
**Product 24**	97 (0.0%)	2,034 (0.0%)
**Product 25**	83 (0.0%)	30,397 (0.0%)
**Product 26**	80 (0.0%)	30,775 (0.0%)
Other products*	379 (0.2%)	318,980 (0.1%)
**Total**	222,119 (100.0%)	351,707,901 (100.0%)

Source: Brandwatch, 2021

CED: Caffeinted Energy Drink

Notes: Percentages may not add up to 100% due to rounding

^a^For Twitter, Reddit, Tumblr, and YouTube.

*Other products includes products 27 to 72

The total number of followers for all Twitter posts mentioning a CED product and the total impressions of each Twitter post for 2020 are shown in Table 2. Overall, the Twitter accounts that have posted on Twitter about a CED product had a cumulative total of 3,635,020,251 followers and had a total of 4,925,122,669 impressions on the CED-related posts. The most popular product was the same as shown in Table 1, accounting for 84.5% of the total followers and 81.7% of the total impressions. Similar to the mentions and reach outcomes, men accounted for the majority of the posts that contained gender information, accounting for 64.2% (212,127,420) of the followers and 66.8% (393,033,438) of the impressions (data not shown).

**Table 2:** Total followers (cumulative) and total impressions of user-generated CED posts on Twitter in 2020

Product name	Total Followers n (%)	Total Impressions n (%)
**Product 1**	3,072,079,528 (84.5%)	4,022,203,966 (81.7%)
**Product 2**	306,073,083 (8.4%)	423,507,723 (8.6%)
**Product 5**	81,095,726 (2.2%)	98,579,299 (2.0%)
**Product 4**	61,538,512 (1.7%)	75,977,587 (1.5%)
**Product 3**	32,669,299 (0.9%)	47,754,378 (1.0%)
**Product 6**	29,028,014 (0.8%)	182,852,842 (3.7%)
**Product 10**	19,549,941 (0.5%)	23,474,437 (0.5%)
**Product 7**	11,038,027 (0.3%)	13,991,169 (0.3%)
**Product 9**	4,649,461 (0.1%)	5,489,301 (0.1%)
**Product 8**	3,412,253 (0.1%)	6,940,535 (0.1%)
**Product 18**	3,094,086 (0.1%)	7,208,274 (0.1%)
**Product 13**	2,691,526 (0.1%)	5,175,694 (0.1%)
**Product 37**	2,082,378 (0.1%)	2,082,705 (0.0%)
**Product 11**	955,739 (0.0%)	1,356,054 (0.0%)
**Product 14**	720,165 (0.0%)	1,443,425 (0.0%)
**Product 12**	673,497 (0.0%)	1,307,955 (0.0%)
**Product 17**	559,781 (0.0%)	1,305,208 (0.0%)
**Product 15**	552,431 (0.0%)	756,588 (0.0%)
**Product 28**	549,559 (0.0%)	641,352 (0.0%)
**Product 20**	487,400 (0.0%)	672,733 (0.0%)
**Product 29**	325,808 (0.0%)	575,103 (0.0%)
**Product 16**	260,961 (0.0%)	397,278 (0.0%)
**Product 19**	217,457 (0.0%)	449,264 (0.0%)
**Product 22**	185,282 (0.0%)	310,993 (0.0%)
**Product 23**	148,590 (0.0%)	152,938 (0.0%)
**Product 30**	135,878 (0.0%)	159,528 (0.0%)
Other products*	245,869 (0.0%)	343,171 (0.0%)
**Total**	**3,635,020,251 (100.0%)**	**4,925,122,669 (100.0%)**

Source: Brandwatch, 2021

CED: Caffeinated Energy Drink

**Notes**: Percentages may not add up to 100% due to rounding.

*“Other products” category includes products 21, 24–27, 31–36, and 38–72.

### Company-generated analysis

At least one valid Canadian social media company-owned account, either on Twitter, Instagram, or Facebook was found for 27 CED brands.

For Twitter, 18 company-owned accounts were identified, and 3 were excluded as these companies do not exclusively sell CED products and did not post any content related to their CED product on Twitter in 2020. Overall, the 15 accounts included in the analysis posted on Twitter a total of 548 times reaching a total of 8,573,781 Twitter users in 2020 (Table 3). There were two main CED brands that posted the most frequently on Twitter and accounted for the greatest reach, together making up 405 (73.9%) of the mentions and reaching an estimate of 5,360,990 (62.5%) users. The third most popular brand accounted for 11.5% of the mentions and 36.8% of the reach, however this company account also posted content related to their non-CED products.

**Table 3:** Frequency of CED company-generated mentions on Twitter and total reach of posts in 2020

Brands	Frequency of Mentions n (%)	Aggregate Reach n (%)
**Brand 1**	204 (37.2%)	2,039,753 (23.8%)
**Brand 2**	201 (36.7%)	3,321,237 (38.7%)
Brand 3*	63 (11.5%)	3,155,696 (36.8%)
**Brand 4**	46 (8.4%)	12,941 (0.2%)
**Brand 5**	20 (3.6%)	N/A**
**Brand 6**	14 (2.6%)	44,154 (0.5%)
Other brands***	0 (0.0%)	0 (0.0%)
**Total**	548 (100%)	8,573,781 (100.0%)

Source: Brandwatch, 2021

CED: Caffeinated Energy Drink

Percentages may not add up to 100% due to rounding

*Data not exclusive to CED products

**N/A: Data was not available by Brandwatch

**Note**: Brands 16–18 are not included as these brands did not advertise a CED product on Twitter in 2020.

***“Other brands” category includes brands 7–15.

The same top two brands on Twitter also accounted for the greatest number of followers and impressions shown in Table 4. Overall, the company-generated posts reached a cumulative total of 17,143,882 followers and had a total of 42,145,939 impressions, with the top two most popular CED brands accounting for 92% of the followers and 81.8% of the impressions.

**Table 4:** Total followers (cumulative) and total impressions of CED company Twitter accounts in 2020

Brands	Total Followers n (%)	Total Impressions n (%)
**Brand 2**	11,171,153 (65.2%)	24,100,762 (57.2%)
**Brand 1**	4,599,714 (26.8%)	10,360,090 (24.6%)
Brand 3*	1,327,813 (7.7%)	7,500,072 (17.8%)
**Brand 4**	25,619 (0.1%)	37,532 (0.1%)
**Brand 6**	19,494 (0.1%)	136,206 (0.3%)
**Brand 5**	11,277 (0.0%)	89 (0.0%)
Other brands**	0 (0.0%)	0 (0.0%)
**Total**	17,143,882 (100.0%)	42,145,939 (100.0%)

Source: Brandwatch, 2021

CED: Caffeinated Energy Drink

Percentages may not add up to 100% due to rounding

*Data not exclusive to CED products

**Note**: Brands 16–18 are not included as these brands did not advertise a CED product on Twitter in 2020.

**“Other brands” category includes brands 7–15.

On Instagram and Facebook, 26 company-owned accounts were identified as having at least one of the two platforms, and 4 were excluded as these companies do not exclusively sell CED products and did not post any content related to their CED product on Instagram or Facebook between July and September 2021. Overall, these company accounts posted a total of 459 times and reached 2,330,743 users on these platforms (Table 5). Only one of the top two CED-company Twitter accounts also had a Canadian company Instagram account. The top account published the most frequently (23.5% of posts) and reached the greatest number of users (81.3% of reach) compared to all other Instagram/Facebook Canadian CED accounts within the 2021 timeframe. When adding up the other top 5 CED brands who posted the most often on these platforms, these brands make up 55.6% of the total mentions, but only reached 10.3% of users.

**Table 5:** Frequency of CED company-generated mentions on Facebook/Instagram and total reach of posts in July-September 2021

Brands	Frequency of Mentions n (%)	Aggregate reach n (%)
**Brand 2**	108 (23.5%)	1,894,796 (81.3%)
**Brand 19**	68 (14.8%)	16,646 (0.7%)
**Brand 20**	58 (12.6%)	69,983 (3.0%)
**Brand 13**	53 (11.5%)	57,134 (2.5%)
**Brand 21**	53 (11.5%)	67,740 (2.9%)
**Brand 11**	26 (5.7%)	30,852 (1.3%)
**Brand 15**	19 (4.1%)	40,879 (1.8%)
**Brand 6**	19 (4.1%)	30,230 (1.3%)
**Brand 22**	14 (3.1%)	7,175 (0.3%)
Brand 16*	12 (2.6%)	38,276 (1.6%)
**Brand 23**	11 (2.4%)	42,187 (1.8%)
**Brand 10**	11 (2.4%)	33,605 (1.4%)
**Brand 8**	6 (1.3%)	1,120 (0.0%)
**Brand 24**	1 (0.2%)	120 (0.0%)
Other brands**	0 (0.0%)	0 (0.0%)
**Total**	459 (100.0%)	2,330,743 (100.0%)

Source: Brandwatch, 2021

CED: Caffeinated Energy Drink

*Data not exclusive to CED products

Percentages may not add up to 100% due to rounding

**Note**: Brands 3, 17, 18 and 27 are not included as these brands did not advertise a CED product on Instagram or Facebook in July, August or September 2021.

**“Other brands” category includes brands 4, 5, 7, 9, 12, 14, 25, 26.

### Content analysis

The frequency of marketing techniques identified on Twitter in 2020 and Facebook/Instagram in July-September 2021 are presented in Table 6. A total of 497 company-generated Twitter posts published in 2020 were included in the marketing technique analysis. Twitter posts contained between 0 and 9 marketing techniques, with an average of 3.9 marketing techniques per post. The most frequently used marketing technique was viral marketing (e.g. hashtags, tagging) and appeared in 82.3% of the Twitter posts. The second most frequently used marketing technique was the presence of teen themes, in particular featuring sports or extreme sports, video games, popular music/culture, and socializing, which appeared in 73.2% of Twitter posts. The most commonly used marketing techniques were followed by cross-promotions (39.6%), calls-to-action online (38.4%) and the presence of a celebrity endorsement (36.2%). Among the posts containing a celebrity endorsement, athletes were the most often endorsing the brand/product and were present in 66.1% of the posts.

**Table 6:** Overall frequency of marketing techniques featured in Twitter and Facebook/Instagram CED company-generated posts in 2020–2021

Marketing Technique	Twitter Frequency January-December 2020 (N = 497) n (%)	Facebook/Instagram Frequency July-September 2021 (N = 424) n (%)
**Audio/Visual Elements**		
**Animation**	85 (17.1%)	34 (8.0%)
**Special effects**	65 (13.1%)	16 (3.8%)
**Music/jingles**	99 (19.9%)	71 (16.7%)
**Child-Related**		
**Presence of children**	4 (0.8%)	4 (0.9%)
**Child language**	1 (0.2%)	0 (0.0%)
**Child themes**	48 (9.7%)	2 (0.5%)
**Licensed characters**	28 (5.6%)	0 (0.0%)
**Parent-child situations**	1 (0.2%)	2 (0.5%)
**Adolescent-Related**		
**Presence of teens**	7 (1.4%)	2 (0.5%)
**Teen language**	28 (5.6%)	4 (0.9%)
**Teen themes**	364 (73.2%)	167 (39.4%)
**Miscellaneous**		
**Cross-promotions**	197 (39.6%)	140 (33.0%)
**Reference to special occasions or holidays**	18 (3.6%)	6 (1.4%)
**Presence of celebrity endorsement**	180 (36.2%)	86 (20.3%)
*Social media influencer*	*4 (2.2%)*	*0 (0.0%)*
*Other celebrity*	*15 (8.3%)*	*11 (12.8%)*
*Musician*	*42 (23.3%)*	*1 (1.2%)*
*Athlete*	*119 (66.1%)*	*74 (86.0%)*
**Purchase Incentives**		
**Contests or giveaways**	30 (6.0%)	4 (0.9%)
**Price promotions**	8 (1.6%)	10 (2.4%)
**Interactive Elements**		
**Call-to-action online**	191 (38.4%)	133 (31.4%)
**Viral marketing**	409 (82.3%)	392 (92.5%)
**Games/polls**	3 (0.6%)	5 (1.2%)
**User-generated content**	21 (4.2%)	44 (10.4%)
**Advertisement Appeals**		
**Convenience**	26 (5.2%)	22 (5.2%)
**Novelty/new**	34 (6.8%)	24 (5.7%)
**Health appeal**	77 (15.5%)	155 (36.6%)

CED: Caffeinated Energy Drink

Notes: For companies that do not exclusively sell CEDs, only CED related posts were coded for marketing techniques

The following techniques were measured but not included in our table/results: Economical, Family appeal, Gender appeal, Spokes characters/spokespeople, Child incentives, Child product

Percentages may not add up to 100% due to rounding

For Instagram and Facebook, a total of 424 company-generated posts published between July and September 2021 were included in the marketing techniques analysis. Posts on these platforms contained between 0 and 8 marketing techniques, with an average of 3.1 marketing techniques per post. As with Twitter, the most frequently used marketing technique was viral marketing and appeared in 92.5% of posts. Again, similarly to Twitter, the second most frequently used marketing technique was the presence of teen themes appearing in 39.4% of posts. The third most common technique was the use of health appeals (36.6%), which was also frequently found in Twitter posts (15.5%), and contained health or nutrition claims such as “sugar free”, “low calorie”, “organic”, “vitamins”, or “all-natural”. These common techniques were followed by cross-promotions (33.0%), calls-to-action online (31.4%) and the presence of a celebrity endorsement (20.3%). Again, athletes were most often endorsing the brand or product on Instagram and/or Facebook and were present in 86.0% of the posts with a celebrity endorsement.

## Discussion

The present study found that CED marketing is prevalent on Canadian social media, and a variety of marketing techniques are used to appeal to diverse social media users, including children and adolescents.

### User-generated results

This study found that social media users frequently mention Canadian CED products when publishing content on Twitter, Reddit, Tumblr and YouTube, with one product being the most widely mentioned, achieved the largest reach, and had the largest a number of impressions. These results demonstrate the popularity of CEDs on social media among peers, as conversations and engagement surrounding CED products are widespread, providing a platform to share information, thoughts and social norms. These peer interactions may contribute to social pressures or normalization surrounding CED consumption, especially as these products are being mentioned in posts by trustworthy sources (peers) [36–38]. As such, the frequent presence of CEDs online among peers may influence CED use, particularly among adolescent social media users, as they are more susceptible to social pressures online and view peers more positively when they post about unhealthy food and beverages [29, 37, 39]. Indeed, adolescents are more likely to adhere to the social norms when seeking social approval through social media or other media, therefore an unhealthy digital environment driven by posts shared by peers may predict actual consumption of unhealthy products, including CEDs [29, 36, 39].

Furthermore, as social media users are posting content related to CEDs and sharing posts with their networks, the users become marketers themselves, without an additional cost for the CED companies [19, 40]. This exponential form of marketing by social media users increases the power and the exposure of the CED marketing and has been successfully leveraged by companies selling unhealthy products [37, 41]. Public education initiatives should be considered to raise awareness about the potential health impacts of a user’s online activity that contributes to the unhealthy CED marketing environment on social media.

### Gender results

This study found that more men (72.5%) than women (27.5%) are posting conversations mentioning CED products and men engage more often with CED-related content on social media. These findings are unsurprising given that children and adolescents perceive CEDs to have gendered branding that targets men, and CED marketing has a greater influence on purchase intention among men than women [42]. These findings are consistent with the literature on the characteristics of CED consumers being mostly men, who are more likely to consume CEDs earlier and more often than women [5, 42–44]. Of great concern is that these gender differences are also observed in CED-related adverse health outcomes and risk-taking behaviours, where men are more likely to mix CEDs with alcohol and more likely to be admitted to emergency care after consuming CEDs with alcohol than women [5, 13, 45].

### Company-generated results

This study found that CED brands are extensively promoting their products on Twitter, Instagram and Facebook and reaching over 10.8 million users on these platforms in 2020–2021. The results show that two leading CED brands have the strongest Twitter presence compared to other CED brands. These findings are similar to US data, where two leading CED brands dominate social media platforms, as well as the majority of the mainstream CED market [46, 47].

Brands use social media to create relationships with consumers and influence attitudes surrounding their products [20, 48]. Although it was not possible to determine the age group of users engaging with these company-generated posts, adolescents report frequent engagement with food and beverage brands, including CEDs, on social media [49]. Youth are also more likely to possess positive attitudes and greater CED purchase intent after being exposed to CED digital marketing [20]. Given this association, adolescents are likely to be primarily exposed to the social media marketing of two major CED brands, which in turn may influence their attitudes towards these products in particular.

### Marketing techniques

This study found that CED companies use an average of 3 to 4 marketing techniques per post on social media, promoting user engagement with posts primarily through viral marketing strategies on Twitter, Facebook and Instagram, which encourages users to interact with their posts by liking, tagging, commenting and sharing. As with user-generated marketing, viral marketing is an important strategy, as peer users contribute to the greater promotion and normalization of CED products. In turn, peer marketing has a powerful influence on consumer attitudes and use of unhealthy products [19, 39].

#### Teen themes

This study found that a significant number of CED company posts contain marketing techniques that may appeal to adolescents, by featuring teen themes such as extreme sports, video games and popular music. Although much less common than adolescent themes, this study also found that 9.7% of Twitter posts also contained child-appealing themes due to the presence of content from child-rated video games.

On Twitter, where two leading CED brands are active, nearly three quarters of the total posts included teen themes, the second most common technique. On Instagram and Facebook, teen themes were also the second most common technique and are featured in almost 40% of the posts. When compared to Twitter, Instagram and Facebook have less posts that feature teen themes, likely because one of the two leading brands on Twitter did not meet the eligibility criteria to be included in the Instagram/Facebook analysis. Although one would think that Twitter is dominated by older age groups, about 32% of 13- to 17-year-olds reported regularly using Twitter in 2018 (compared to 51% using Facebook and 72% using Instagram). Teen presence on Twitter coupled with the presence of teen themes in CED posts therefore raises a health concern [29]. These findings suggest that CED companies are directly targeting adolescents on social media, a group that is already vulnerable to the influential power of marketing [50, 51].

In the US, CED advertisements are most often featured on programs with adolescent themes such as extreme sports and popular music [52]. These findings also reinforce the image that CED brands often portray as being cool and risky [53]. Other sugar-sweetened beverages use similar teen themes in their social media campaigns, particularly when advertising to teens and adults [47, 50, 53]. While other sugar-sweetened beverage brands also frequently market to children using child-appealing themes such as fun graphics and cartoon images on social media, US CED brands were found to have the highest social media presence using sponsored music, extreme sports, and gaming to promote their products to teens, exceeding all other sugar-sweetened beverages on Twitter, Instagram, and YouTube in 2019–2020 [47, 53, 54]. Overall, these findings are consistent with this study, as well as the literature that indicate that CEDs are often consumed by adolescents during sports and when computer gaming [2, 43]. Sport-related themes in CED advertisements also contribute to the perception that these products are targeting younger audiences and are to be consumed during sports [2, 4]. However, CED use during sports is potentially harmful, especially if used for hydration [23].

In addition to having a profound effect on shaping child behaviours and attitudes through peer and brand influence, Canadian children and adolescents are often online and use social media for several hours a day, making them likely to be exposed to CED marketing [19, 20, 30, 38, 39, 62]. In Canada, about one-third of children under 13 reported having a social media account (even though it is not permitted by most platforms) and over 55% of children between 10 and 13 years old reported owning a smartphone [26, 28, 34, 55]. Although it was not possible to assess age-specific exposure in this study, previous research has found that Red Bull (a popular CED) was among the top 5 most frequently advertised food or beverage products on preferred websites of Canadian children between 2 and 11 years old [26]. Previous research also found that Red Bull was the third-most frequently advertised food or beverage product on adolescent (12–17 years old) websites [56]. Though targeting adolescents is not prevented by current Canadian CED policies, exposure to CED marketing that appeals to teens has important health implications in this population, as it may increase the attitudes, preferences and consumption of these harmful products and subsequent adverse health effects [11, 37].

#### Cross-promotions

Cross-promotion strategies are frequent on Twitter (39.6%) and Instagram/Facebook (33.0%). As observed in other studies, CEDs use cross-promotions to likely appeal to a greater variety of audiences by connecting with consumers of other brands, particularly fast-food, video games, and events (generally sporting, extreme sporting, artistic or music events) [46, 57]. The present study also found that CED brands were promoting the free distribution of their products at local cross-promotion events to encourage consumption of their products, a technique that is frequently used in other countries as well [42, 58]. CED brand presence at sport or family-friendly events would be a concern in cases where consumption is encouraged [35].

#### Celebrity endorsements

Another frequent strategy is the use of celebrity endorsements, which was found in 36.2% of Twitter posts and in 20.3% of Instagram and Facebook posts, primarily featuring athletes (66.1% and 86.0% for Twitter and Instagram/Facebook, respectively). These findings are consistent with a US study that found a high prevalence of athlete sponsorships in energy drink and soda ads [3]. Athlete celebrity endorsements are especially influential to pre-adolescent boys, who are more likely to choose a product if it features a sports celebrity and perceive the endorsed product to be healthier [59].

#### Health appeals

Health appeals were found in 15.5% of Twitter posts and 36.6% of Instagram/Facebook posts. Posts using this marketing technique include words or phrases such as “low calorie”, “sugar free”, “all natural”, “added vitamins”, “organic” or general reference to the nutritional content of the CED, as exemplified by the following Instagram post analyzed in this study: “*Made with 180 mg of caffeine, B vitamins, no sugar, and no calories so you can sip #GuiltFree*!”. The use of health appeals is concerning given the adverse health effects associated with CED consumption, in addition to contributing to the normalized perception that CED consumption is a safe, or even healthy behaviour [19, 60, 61]. This marketing strategy is considered “health-washing” a product, defined as “presenting genuinely unhealthy products in a misleading context […] related to a healthy lifestyle.” [62]. Health-washing may influence parents and older children to perceive certain products are being healthier than they are [63, 64]. The appropriateness of continuing to allow any health claims associated with CEDs should be assessed by governments, as these nutrition-related claims may mislead consumers and effectively influence CED consumption [59, 62, 63]. Health Canada permits health claims if they are supported by evidence and linked to a specific substance in the product [23]. Claims that are too general, related to sport performance, imply good health, or that refer to the product as a source of nutrients are not permitted [23]. Certain health appeals found in this study may create the impression that CEDs are needed for a healthy lifestyle or as an essential a source of vitamins. These claims are inappropriate as they mislead consumers and dismiss the potential health risks of CEDs. As such, the use of health appeals in Canadian social media posts likely go against Health Canada’s CED regulations and compliance checks are required [23].

### Policy implications

Under Health Canada regulation and voluntary restriction of CED marketing by the Canadian Beverage Association, it is prohibited to market CEDs to children under 12 years old [23, 24]. However, there are significant regulatory gaps surrounding these restrictions.

This study highlights the extent of CED marketing on social media, where Canadian children are likely to be exposed due to their frequent social media use, signalling the weaknesses of current policies to restrict CED marketing to children. Alarmingly, social media marketing of CEDs is not restricted under current regulations, despite previous evidence that children are frequently on social media and have been exposed to CED marketing online [26, 28, 34, 55]. This study also identified instances of child-appealing themes on CED social media accounts, suggesting that children may be targeted by certain CED marketing. As such, although children are officially protected, the compliance with self-regulatory policies and with marketing restrictions imposed by Health Canada may be low and current regulations should likely be strengthened, monitored, and enforced. Potential models to examine include Sweden, Latvia, and Lithuania that limit advertisement and sale of CEDs to adolescents [21].

Further, current Health Canada regulations fail to specify the marketing settings, audience thresholds, and marketing techniques when restricting the exposure of CED marketing to children. CED marketing restrictions should be expanded to prohibit the use of child-appealing strategies and marketing within the digital media space, which would require global efforts and systematic monitoring to ensure that regulations are effective at protecting children.

Another concerning gap in Canadian CED regulations is the omission of adolescents, despite formal recommendations to limit the consumption of CEDs to adults over 18 years old in Canada due to their adverse effects [23, 65]. This study found that CED marking is widespread on social media and the use of themes to appeal to adolescents by CED brands is highly prevalent. To protect adolescents between the ages of 12 and 18 years old, policy action must be considered to address the frequency and content of CED social media posts, particularly those containing adolescent-targeting strategies, which have a powerful impact on their attitudes, consumption, and associated health risks [16, 20, 37, 65, 66]. The findings of this study may help inform future regulatory decision-making to include more stringent restrictions surrounding CED social media marketing in Canada to protect children and adolescents.

### Strengths and limitations

To our knowledge, this is the first study to assess user-generated and company-generated CED social media posts in Canada using online licensed data from a social media monitoring company, and to assess the marketing strategies used by Canadian CED brands on social media. The limitations of this study are largely due to the nature of the licensed Brandwatch data. First, it was not possible to collect age-specific information or exposure data. Second, non-Canadian posts may be captured in the user-generated data, as Brandwatch requires geotags to filter mentions by locations, and not every post contains a geotag, which likely increases the number of posts. Third, it is possible that the frequency of mentions for certain products may be underestimated due to the inability to write queries that capture all mentions, while simultaneously avoiding capturing irrelevant mentions and remaining methodologically consistent. Queries that may be too specific for a given product may underestimate the total number of mentions. Fourth, Brandwatch does not capture posts with product mentions contained in photos or in videos (e.g., product placements by influencers), and only collects text data. Therefore, the findings likely underestimate the true number of mentions if users frequently post pictures or videos of CEDs on social media. Finally, Brandwatch does not detect or differentiate between real and fake accounts, such as bots, that may potentially inflate the data [67].

Other limitations of Brandwatch pertain to the differences in methodologies between Twitter, Instagram, and Facebook. Although it was possible to collect historical data for Twitter for all of 2020, it was only possible to collect Instagram and Facebook data for three months in 2021, which limits comparability across platforms. Additionally, seasonal bias may have been present, as beverage companies may post more frequently during the summer months, which may limit the generalizability of the findings on Instagram/Facebook which were only assessed from July to September 2021.

Further, only Canadian accounts were included in this study to keep results relevant to Canada. However, American accounts are generally more popular and post more often than Canadian accounts, and likely reach Canadian social media users, which may not have been captured in this study.

The brands in the company-level analysis were not the same for Twitter, Instagram, and Facebook, which limits comparability across these platforms. Certain brands did not have social media accounts on all three platforms, while other brands (particularly non-Canadian brands) had valid social media accounts, however they were not Canadian.

Lastly, we defined engagement as the number of impressions (retweets, searches, and views) and followers on Twitter posts. According to the four levels of the engagement framework by Shawky et al. (2021) (*connection, interaction, loyalty, and advocacy*) our study assesses the measures of connection and interaction only [68]. As such, our findings may not capture the complexities of social media engagement, as it does not account for the multi-actor social media framework [68].

## Conclusion

This study found a significant number of CED posts on social media in 2020–2021 and many of these posts have marketing techniques that may appeal to adolescents. Despite current CED regulations, children and adolescents are likely to be exposed to CED marketing on social media which in turn may influence their preference and consumption behaviors. High levels of caffeine, sugar, and other active ingredients in CEDs, pose a risk of experiencing adverse health effects if consumed by younger populations. Canadian policymakers should consider the prevalence of CED marketing on social media when designing improved CED marketing restrictions to protect the health of children and adolescents.

## Data Availability

The data generated and analysed during this study are available from the corresponding author on reasonable request.

## List of Abbreviations

CBA: Canadian Beverage Association
CED: Caffeinated Energy Drink
FDA: Food and Drugs Act
US: United States
